# Misconceptions on Grief and Bereaved People’s Needs Among Health and Social Care Professionals, Teachers and General Population in Italy

**DOI:** 10.3390/bs16030420

**Published:** 2026-03-13

**Authors:** Claudia Venuleo, Simone Rollo, Daniela Nogueira

**Affiliations:** 1 Department of Human and Social Sciences, University of Salento, 73100 Lecce, Italy; 2 Department of Humanities, University of Foggia, 71121 Foggia, Italy; simone.rollo@unifg.it; 3 Department of Behavioral and Social Sciences, ISMAI—Instituto Universitário da Maia, Porto District, 4425 Maia, Portugal; dnogueira@umaia.pt

**Keywords:** myths, grief, health and social care professionals, teachers, general population, training needs

## Abstract

This study investigated misconceptions about grief among health and social workers, teachers, and the general population in Italy. Socio-demographic characteristics, including gender, age, education, and personal experience of loss, were also examined. A questionnaire with nine statements capturing common myths about grief and its management was completed by 233 participants (mean age = 42.2, SD = 12.7; 60.9% women) using a 4-point Likert scale. Results showed that most participants responded inaccurately to most statements, agreeing with the idea that grief proceeds in a linear fashion, overlooking the heterogeneity of grief reactions, pathologizing them, and overestimating the need for psychological support. Health and social workers provided more accurate responses than teachers and the general population for certain myths, particularly regarding grief in children and older adults. Significant differences were also observed for some myths in relation to gender and whether participants had experienced a significant loss. These findings highlight the widespread endorsement of grief-related misconceptions and the compelling need to improve public awareness of the grieving process. They provide valuable insights into the key topics that should be addressed when designing initiatives to enhance community competence in supporting bereaved individuals.

## 1. Introduction

Grief is a universal human experience but, at the same time, a highly idiosyncratic process. Although some emotional, cognitive, behavioral, and physical reactions, as well as existential thoughts, are common, the way in which each person reacts to a major loss depends on a multiplicity of factors. These factors include the nature of the death, the quality of the relationship with the deceased, attachment patterns, life history, the age of the bereaved, the support received, cultural context, and historical circumstances.

Just as there is no single way to react to a loss and manifest one’s grief, there is also no single set of needs among the bereaved, nor a single type of help that can be offered to them. According to the “Grief Pyramid,” a model inspired by the evidence-based public health approach to bereavement care, as advanced by NICE (UK), Aoun et al. (2017, 2018, 2019) and the Irish Hospice Foundation (URL: https://bereavement.eu/, accessed on 5 December 2025), bereaved individuals range in their needs from basic information and support to a high level of specialized care.

To highlight the importance of a tailored response, research-based estimates suggest that around 10% of bereaved individuals experience grief that remains intense and debilitating over a long period, requiring professional mental health intervention (tier 3 of the Pyramid); approximately 40% experience a difficult healing process and require additional psychosocial support (tiers 2 and 3); while most people undergo a natural and healing grieving process and only need basic information and general support (tier 1).

However, there is a tendency in modern Western society to treat bereavement as a psychiatric or psychological problem (Breen et al., 2022; Granek, 2008), “located within” the individual, and to consider the responsibility of ensuring adequate support primarily a matter for mental health professionals (Zuniga-Villanueva et al., 2021). A recent qualitative study (Marinaci et al., 2024), based on 162 in-depth interviews with grievers’ informal support providers (relatives and friends, teachers, religious leaders, funeral providers, pharmacists, volunteers, and social service workers), revealed that respondents tend to underestimate the help they could offer to the bereaved and to emphasize the need for psychological or medical intervention, as if grief over the death of a loved one could not be accepted as a natural response to loss and had to be immediately ‘medicated’ or otherwise processed and resolved.

This increasing tendency to medicalize and individualize grief can be better understood within the wider socio-cultural transformations that have reshaped the experience of death and mourning in contemporary Western societies. Historically, collectively shared cultural frameworks structured mourning practices by providing widely recognized rituals, social norms, and expectations regarding how bereavement should be expressed and supported (Ariès, 1981; Walter, 1994; Rosenblatt, 2008). Mourning was often embedded in communal and religious practices such as home vigils, funeral rites, memorial masses, and visits of condolence, while social conventions regulated visible signs of grief, including wearing black clothing, observing specific mourning periods, or temporarily withdrawing from certain social activities (de Martino, 1977; Seale, 1998). In contexts such as Italy, where mourning practices were traditionally strongly embedded in family and community life, these rituals also contributed to publicly acknowledging the status of the bereaved and mobilizing collective forms of support.

During the twentieth century, processes such as secularization—that is, the declining influence of religious beliefs and institutions in shaping social norms and practices—together with the medicalization of dying and the increasing privatization of death, have progressively weakened these shared cultural frameworks that once structured mourning practices and provided socially recognized roles for the bereaved (Ariès, 1981; Walter, 1994, 1996). As discussed by Walter (1994), when culturally transmitted knowledge about how to respond to loss becomes less visible or less collectively regulated, individuals may increasingly rely on simplified psychological narratives to interpret grief. In Italy, anthropological and sociological research has documented similar shifts: the professionalization of funeral rites and the attenuation of communal participation in mourning and the progressive displacement of death from the home to hospitals have reduced opportunities for the transmission of shared cultural knowledge about grief (Colombo & Molinari, 2022; de Martino, 1977). For instance, historical data show that while in the early 1900s most deaths occurred at home, by the late twentieth century a majority of deaths—especially in northern and central regions—occurred in hospital settings, limiting families’ direct experience with end-of-life care and mourning rituals (Colombo & Molinari, 2022). In this context, popularized interpretations of psychological models may contribute to the persistence of beliefs that grief is not only a predictable and uniform process, but also an individual rather than a socially and communally embedded experience.

Embracing the compassionate community perspective (Aoun, 2022; Hilbers et al., 2018; Rumbold & Aoun, 2014) and the core idea that grief is everyone’s responsibility (Aoun, 2022; Kellehear, 2013), we emphasize the view that how bereaved people react and cope with loss also depends on how grief is perceived, discussed, and managed at a societal and collective level. The COVID-19 pandemic clearly highlighted how the lack of adequate support, due to the isolation imposed by lockdown during the most acute phases, posed a major threat to the mental health of bereaved family members, making them vulnerable to complicated grief reactions (Mortazavi et al., 2023). More generally, the quality of support received from the community, workplace, school, or other civic networks can make a difference in the likelihood that bereaved people feel isolated and powerless (Burke & Neimeyer, 2014; Cacciatore et al., 2021; Stroebe & Schut, 2005, 2021; Venuleo et al., 2026). Previous studies have suggested that social support functions as a mediator for proactive coping in grief (Rogalla, 2020) and as a means to mitigate both the intensity and duration of psychological distress and negative physiological outcomes (Aoun et al., 2018; Burke & Neimeyer, 2013).

Much of the support for the bereaved is provided by people already involved in their everyday lives, such as family and friends (Aoun et al., 2019; Venuleo et al., 2026), but there are also many other community actors who—because of their professional roles (e.g., teachers, nurses, funeral providers, priests, police officers)—can play a crucial role in supporting bereaved people (Venuleo et al., 2022). In particular, in this study, we emphasize the strategic role of non-mental health and social professionals working in hospital and nursing home settings, as well as teachers. Both of these professional categories, in their different functions and operational contexts, can offer support at strategic moments in the bereavement process. The former potentially accompany family members at the end of the inpatient’s life, are present at the time of death, and can ensure the bereaved family members’ needs are met in the immediate aftermath (Raymond et al., 2017; Wimpenny & Costello, 2013). Teachers have an ongoing relationship with bereaved pupils and can play a potentially strategic role by preparing the class to welcome the bereaved student back to school, providing opportunities for children to discuss what has happened, supporting bereaved pupils’ adjustment in the months following the loss, and opening dialogue and reflection on loss and bereavement as a potentially integral part of the school curriculum (Dawson et al., 2023; Dimery & Templeton, 2021).

Non-mental health support providers often report low levels of confidence in handling grief and are afraid of giving inappropriate responses (Dodd et al., 2022; Galende, 2015; Marinaci et al., 2024; Testoni et al., 2021). Myths and misconceptions related to grief and its expression can be an obstacle to considering themselves competent agents in offering support and may actually lead to inappropriate attitudes towards the bereaved, fuelling their distress or making them feel lonelier (Bonanno, 2004; Wortman & Boerner, 2011; Wortman & Silver, 2001). For example, the idea that a healthy bereavement requires people to quickly regain control over their lives can make individuals feel that they are weak, defective, or sick if they are unable to do so. Similarly, the belief that people become depressed after a major loss may lead bereaved individuals to think they are experiencing the wrong emotions or coping differently from what their network suggests as “normal.” This additional pressure to grieve in a particular way can also lead to increased distress and, consequently, worse outcomes, as seminal work by Wortman and Silver (1989) has shown.

Wortman and Silver (1989) described several common myths that are not supported by empirical research—for example, that distress or depression is inevitable, that failure to experience distress indicates pathology, that bereaved individuals must work through a loss, that there is a final stage of grief related to recovery, and that a state of resolution or acceptance is achieved after working through a loss. In a follow-up article, Wortman and Silver (2001) noted that “these myths are still prevalent among clinicians and the general public” (p. 424). In a study examining the prevalence of coping myths (e.g., “People go through predictable stages of different emotions after experiencing trauma,” “People who do not experience negative emotions shortly after a traumatic event will develop emotional problems later on,” and “People who do not find resolution or acceptance after a traumatic event will experience prolonged grief”) in a large representative sample of Americans, Tatar (2017) found that over 60% of participants reported at least moderate endorsement of coping myths, with just over 10% reporting strong endorsement. Sawyer et al. (2022) examined misconceptions about grief and bereavement in a sample of US mental health professionals and the general public. They found that mental health professionals responded accurately to most statements (e.g., it was widely acknowledged that most people do not develop a mental disorder after a loss or require professional help to cope with grief, and that children grieve just as deeply as adults and should not be shielded from the pain caused by death). The general public, on the other hand, answered just over half of the statements correctly (e.g., the majority incorrectly assumed that stage theories of grief are accurate, overestimated the number of people who develop complicated grief reactions after losing a loved one, and assumed that grief responses are consistent across cultures). Both groups mistakenly believed that grief progresses through predictable stages and overestimated the percentage of people who experience complicated grief. Wortman and Boerner (2011) note that a review of textbooks commonly used in the training of physicians and nurses suggests that these texts often perpetuate coping myths, for example, by maintaining that people go through stages of emotional response as they cope with loss and that failure to show distress indicates a problem. Unfortunately, few scholars have collected data on the endorsement of myths about grief and its management among health and social care professionals, teachers, and the general population. This kind of knowledge could inform training initiatives to improve grief literacy in the community and enable (non-mental health) support providers to offer more effective support to the bereaved—a need that has become even more evident in the aftermath of the COVID-19 pandemic.

Within the framework of the European project Erasmus+ Agency “AURORA@COVID19-EU: Articulating a Unified Response to the COVID-19 Outbreak. Reconstruction After loss in Europe”, aimed at developing guidelines and training activities to increase bereavement support skills in the wider community, the present study aimed to investigate myths and misconceptions on grief among three groups of informal support providers—relatives and friends, teachers, and health and social workers—in the Italian context. To the best of our knowledge, this represents the first study conducted in Italy to investigate grief-related myths and misconceptions.

Given the paucity of available results on how adherence to myths may vary by socio-demographic characteristics, the study also aimed to explore whether age, gender, level of education, and having experienced a significant loss make a difference in how grief is viewed.

## 2. Materials and Methods

The study is part of a mixed-methods, quantitative–qualitative research project aimed at identifying the training needs of informal support providers in Italy in order to better design and tailor future skills training programs that enhance their capacity to offer effective supportive care. In the first phase of the research, informal support providers were asked to recount an experience in which they directly supported a bereaved person. In the second phase—which is the focus of this study—participants completed an online survey including nine statements addressing common myths about grief and its management.

### 2.1. Instrument

An online questionnaire was constructed using Microsoft Forms and nine myths were assessed. Participants were asked to rate their agreement on a 4-point agree-disagree scale (1 = definitely true, 2 = probably true, 3 = probably false, 4 = definitely false).

The myths were selected based on previous work by Sawyer et al. (2022) on a sample of mental health professionals and the general public. These myths are also cited in prior literature (e.g., Jordan & Neimeyer, 2003; Pearce, 2019; Silver & Wortman, 1980; Venkatesan, 2022; Wortman & Boerner, 2011). In the following, the reasons why they are considered false beliefs or misconceptions are briefly outlined.

*Myth 1: “Grieving can be expected to progress through a predictable series of stages, beginning with denial and ending with acceptance.”*

This myth is consistent with traditional grief literature that conceptualizes grief in terms of a series of stages to be passed through, with a beginning and a short-term end, and views it as a pathological process if continued (Breen & O’Connor, 2007, p. 201). Stage theories include John Bowlby’s grief phase theory (Bowlby, 1980), Elisabeth Kübler-Ross’s five stages (Kübler-Ross & Kessler, 2014), and Johan Cullberg’s four phases (Cullberg, 2008), and Other influential frameworks, such as James William Worden’s task model (Worden, 2018), conceptualize bereavement in terms of psychological tasks rather than stages, although they have sometimes been interpreted as implying a sequential progression. However, the 1990s saw a paradigm shift in the understanding of grief. Phase and task models have been criticised for being too schematic in their view of grief. Not all bereaved individuals go through the stages or address the tasks in the suggested order (Tølbøll et al., 2022).

Against a view of grief as a linear process, the Dual-Process Model of Coping with Bereavement suggests a more dynamic perspective (Stroebe & Schut, 2001, 2010), conceiving grief as a regulatory coping process of oscillation. The grieving individual at times engages in loss-oriented processes, relating emotionally and cognitively to the recognition of the irreversible relational loss; at other times, in restoration-oriented processes, they pause the painful emotions and focus on problem-solving in response to concrete life changes (e.g., learning new skills for tasks previously managed by the deceased, changing roles, finding new confidants, or strengthening other relationships). Both processes contribute to adaptive healing, and so-called complicated grief reactions can be understood as resulting from a lack of oscillation between them. Current trends recognise that the array of grief reactions is diverse and complex, and thinking of bereavement as a linear process can lead to pathologizing any reaction that deviates from expected expressions of grief (Doka, 2016).

*Myth 2: “People who do not become depressed after the death of a loved one are likely to denying their true feelings.”*

This myth assumes that depression is the most frequent and natural reaction to a loss and that there is only one way to express one’s grief. However, it overestimates the prevalence of depressive symptoms following a loss. Although some studies show that most (but not all) bereaved individuals experience distress in the months following the death of a significant other, only a minority suffer from clinical levels of depression (Aoyama et al., 2018). Feelings such as sadness and dysphoria are more frequent among bereaved individuals than among non-bereaved individuals, but they do not necessarily reach the intensity associated with depression (Horwitz & Wakefield, 2007), and the majority of bereaved individuals cope adequately with their grief (Lundorff et al., 2017). Researchers have also examined a bereavement-specific syndrome characterized by prolonged and impairing grief, the so-called prolonged grief disorder (PGD); however, it has been estimated that only 10–20% of bereaved adults confronted with natural loss will develop PGD (Lundorff et al., 2017). Other authors have suggested that even the failure to experience distress should not be considered indicative of pathology (Brinkmann, 2018), and that there are many possible explanations for why a bereaved person may not exhibit intense distress (e.g., early adjustment following an expected loss or relief that the loved one is no longer suffering).

*Myth 3: “Most people develop a mental disorder after the death of a loved one.”*

This myth conveys a medicalised and pathological view of grief reactions. As previously mentioned, studies suggest that most people experience a natural and healing grieving process and only need basic information and general support, whereas about 10–20% of people develop chronic and debilitating grief reactions after the death of a loved one, requiring professional help (Aoun et al., 2017, 2018, 2019).

*Myth 4: “Pain responses are typically consistent even when cultural differences are considered.”*

How one reacts to death varies greatly across historical eras, cultures, and societies (Jacobsen & Petersen, 2020; Klass & Chow, 2021; Silverman et al., 2021). For example, while in the Western world it is usual to view depressive states following life events such as bereavement, in most other cultures, somatic symptoms predominate to a far greater extent (Stroebe & Schut, 1998). In studies by Chow et al. (2006), Hong Kong widows claim that they tried to contain their emotions at the time of their husband’s death, for fear that openly expressed emotions would disturb the deceased’s transition to the new world; Chinese families often avoid discussing death and dying, primarily due to fear of invoking bad luck (Hsu et al., 2009). The duration and expression of grief are also shaped by religion and culture. For instance, in Islam, grief expression is limited to the first few days after death, to prevent attachments of the living from hindering the soul’s journey (Wikan, 1988).

*Myth 5 and 6: “Elderly people are usually more anxious about death than young people” and “Bereavement children and adolescents do not grieve as deeply as adults”*

Both statements emphasize the role of age over other factors (e.g., personality, nature of the bond with the deceased, support network), thus underestimating intra-group differences and the significance of younger individuals’ grief reactions, who may consequently be overlooked by support measures (Morris, 2013). Although early theorists, such as Wolfenstein (1966), suggested that grieving does not occur until adolescence due to younger children’s psychological development and underdeveloped object relations, others have documented very young children’s reactions to the loss of a loved one, including protest, despair, and detachment (Bowlby, 1980). Today, scholars widely recognize that the death of a close person (e.g., a parent) is a major stressful event for children, potentially leading to severe psychological and social distress if they are not supported during the acute phase of grief (Zdankiewicz-Ścigała et al., 2019; Mallon, 2011). Like adults, children grieve, even if—as infants and young children—they may not understand the cause or finality of death (Venkatesan, 2022). Children may express grief in a variety of ways (Parkes, 2013; Pearlman et al., 2010), including emotional reactions (e.g., crying more than usual, irritability, anger, or unrealistic guilt for causing the death), physical reactions (e.g., sleeping more than usual, complaining about aches or pains) and behavioural reactions (e.g., isolating themselves from family and friends, or adopting disruptive behaviours). Children may also quickly resume daily routines or appear unaffected, but this does not necessarily indicate rapid recovery; rather, they are seeking alternative ways to cope with their feelings and may require additional emotional space to process them (Koehler, 2010).

*Myth 7: “Generally, it is more useful to ‘move on’ with one’s life rather than thinking about the memories of the deceased.”*

This myth—which encourages people to prematurely distance themselves from their grief rather than approach it, internalising society’s message that mourning should be quiet, quick, and efficient (Wolfelt, 1999)—may originate from Freud (1917), who in *Mourning and Melancholia* suggested that the mourning process involves detachment from the emotional energy invested in the deceased. Although influential, this view has been reconsidered by contemporary grief theories. When people maintain a connection with the deceased despite their physical absence, they construct healthier meanings of the loss and can adapt more effectively to grief (Neimeyer, 2006). Accordingly, bereaved individuals do not need to emotionally detach from their loved one; rather, maintaining a living inner connection with the deceased facilitates grief processing and adjustment to the loss (Guldin, 2019).

*Myth 8: “Experts generally recommend that children be protected from the pain and suffering created by death.”*

This myth conveys the assumption that pain and suffering are avoidable experiences for the youngest and that protecting them involves not sharing feelings within their proximal network. Although this belief appears to be socially widespread (Anderson, 2020; Aspinall, 1996), and many parents believe they are protecting children by avoiding conversations about death (Zdankiewicz-Ścigała et al., 2019), the literature identifies several compelling reasons to encourage parents, teachers, and healthcare professionals to engage in open discussions about illness and death with children (Burton, 2022; Dalton et al., 2019). Children—despite their tender age—are aware of what is happening around them and possess the emotional and, as development progresses, the cognitive capacities to experience the sadness associated with loss, absence, and changes in family structure. Children who are helped to express their needs and who receive support from close others are more capable of building a coherent narrative about their experience of loss, which is widely recognised as a key healing factor in the grieving process (Zdankiewicz-Ścigała et al., 2019).

*Myth 9: “Most people need professional help to deal with pain.”*

Although this myth aligns with the modern tendency to medicalise grief, there is no evidence that uncomplicated grief requires formal treatment or professional intervention (Aoun et al., 2018; Jordan & Neimeyer, 2003). Most bereaved individuals reach an acceptable level of adjustment to life without their loved one without professional help. Their social network—such as family members, friends, teachers, and healthcare professionals—can play a crucial role in the grieving process by offering empathetic listening and validating the bereaved individual’s reactions (Raymond et al., 2017).

Each questionnaire was preceded by a consent form providing detailed information about the study, followed by a socio-demographic section where participants indicated their age, sex, and education level, and stated whether they had experienced a significant loss in their lives.

### 2.2. Recruitment Procedure

Participants were recruited using a combination of purposive and snowball sampling (Naderifar et al., 2017; Tongco, 2007). Initially, interviewers identified potential informal support providers within their own social networks, including both relatives or friends of bereaved individuals and professionals who had offered support in their roles, such as teachers and health and social care workers (purposive sampling). Participants identified were subsequently asked to refer others who had similar experiences supporting bereaved individuals, thereby expanding recruitment through their networks (snowball sampling).

The survey link was then sent to participants who consented to take part in the study.

The study was approved by the Ethics Committee for Research in Psychology (CERP) of the Department of Human and Social Sciences at the University of Salento (protocol No. 78717 of 25 May 2022).

### 2.3. Participants

A total of 233 questionnaires were collected (mean age = 42.2 years, SD = 12.7; 60.9% women). Of these, 87 participants (37.3%) were health and social care workers (including social workers, nursing home coordinators, and hospital nurses), 84 (36.0%) were teachers, and 62 (26.6%) belonged to the general population. Table 1 presents the socio-demographic characteristics of the participants, disaggregated by the three groups.

### 2.4. Data Analysis

Descriptive analyses (i.e., frequencies) were conducted to summarize participants’ responses. Response accuracy was determined by calculating the percentage of participants who endorsed each statement in a manner consistent with current research evidence; accordingly, responses of *probably false* or *definitely false* were considered accurate for each of the nine myths.

Chi-square tests were performed to examine differences across respondent groups (health and social care professionals, teachers, and the general population) and according to socio-demographic characteristics (sex, age, educational level, and whether participants had experienced a significant loss). For this purpose, response categories were collapsed as follows: *probably false* and *definitely false* were grouped as accurate responses, whereas *probably true* and *definitely true* were grouped as inaccurate responses.

All analyses were performed using JASP, Version 0.19 (JASP Team, Amsterdam, The Netherlands, 2026; https://jasp-stats.org), Apple Silicon.

## 3. Results

Table 2 presents, for each myth, the distribution of responses across the three groups considered. Responses that, based on recent literature, are considered accurate (i.e., ‘definitely false’ and ‘probably false’) are highlighted in bold. As shown, respondents across all groups tended to rate most of the proposed statements as *true* or *probably true*.

Considering both the overall sample and each group separately, the majority of respondents provided inaccurate answers (i.e., rated the statements as *definitely true* or *probably true*), with the exception of Myth 3, *“Most people develop a mental disorder after the death of a loved one”*, and Myth 6, *“Bereaved children and adolescents do not grieve as deeply as adults”*, which were correctly identified as false by 63.1% and 55.4% of the overall sample, respectively. Significant role-related differences were found for Myth 5, *“Elderly people are usually more anxious about death than young people”* (χ^2^ = 7.405, *p* < 0.05), with health and social care professionals providing more accurate responses than teachers and the general population, and for Myth 6, *“Bereaved children and adolescents do not grieve as deeply as adults”* (χ^2^ = 12.384, *p* < 0.01), with health and social care professionals and teachers providing more accurate responses than the general population (Table 3).

Table 4 reports the results of the chi-square analyses examining differences in myth endorsement according to participants’ socio-demographic characteristics. Significant sex-related differences were found for Myth 1, *“Grieving can be expected to progress through a predictable series of stages, beginning with denial and ending with acceptance”* (χ^2^ = 7.277, *p* < 0.05), and Myth 6, *“Bereaved children and adolescents do not grieve as deeply as adults”* (χ^2^ = 7.526, *p* < 0.05). Women provided less accurate responses than men for Myth 1, whereas men provided less accurate responses for Myth 6.

Significant differences related to having experienced a significant loss were found for Myth 2, *“People who do not become depressed after the death of a loved one are likely to be denying their true feelings”* (χ^2^ = 5.499, *p* < 0.05), Myth 3, *“Most people develop a mental disorder after the death of a loved one”* (χ^2^ = 3.991, *p* < 0.05), and Myth 7, *“Generally, it is more useful to ‘move on’ with one’s life rather than thinking about the memories of the deceased”* (χ^2^ = 4.119, *p* < 0.05). In all three cases, respondents who had experienced a significant loss provided more accurate responses than those who had not. No significant differences were found with respect to age group or educational level.

## 4. Discussion

This study aimed to investigate misconceptions about grief among (non-mental health) health and social care professionals, teachers, and the general population. To the best of our knowledge, this is the first study to address this topic in the Italian context.

The findings indicate that the nine proposed myths about grief and its management are widely endorsed by participants. Considering both the overall sample and each of the three groups separately, most respondents provided inaccurate answers, with the exception of Myth 3, *“Most people develop a mental disorder after the death of a loved one,*” and Myth 6, “*Bereaved children and adolescents do not grieve as deeply as adults.*” Overall, these results offer a valuable perspective on the key topics that should be addressed when designing initiatives aimed at increasing community competence in providing bereavement support.

The belief that grief unfolds through a predictable sequence of stages—“beginning with denial and ending with acceptance”—endorsed by 94.8% of the sample, is consistent with findings reported by Sawyer et al. (2022), who found that the majority of their general population sample also considered this statement accurate. Stage theories have long dominated the conceptualization of grief and have been widely disseminated through grey literature and social media, often in simplified and radicalized forms. However, this mistaken view of grief as a linear process of “recovery” can lead to the problematization of normal grief reactions (Pearce, 2019), as well as to ineffective support and unhelpful responses from medical professionals (Poxon, 2023). In contrast to a linear conceptualization of grief, familiarity with more recent models that adopt a dynamic perspective—such as the Dual Process Model of Coping with Bereavement (Stroebe & Schut, 2001, 2010)—could provide informal support providers with interpretative frameworks to better understand the grieving process. This model highlights how both loss-oriented processes, in which bereaved individuals emotionally and cognitively confront the irreversibility of the relational loss, and restoration-oriented processes, in which they address life changes, new roles, tasks, and relationships, jointly contribute to adaptive adjustment to loss. Such knowledge may also foster recognition that, even when bereaved individuals have gradually adapted to life without the deceased, they may continue to experience intense feelings of longing and remembrance—for example, on anniversaries or holidays—and that these moments should be understood as natural and physiological rather than as signs of deterioration or “worsening”. This perspective may, in turn, help prevent the medicalization of bereavement reactions and reduce the tendency to prescribe—or to expect the prescription of—sedative medication in response to the most painful emotions associated with loss-oriented moments (Horwitz, 2019).

The belief that there is only one “right” or “normal” way to grieve—such that, for example, individuals who do not become depressed after the death of a loved one are assumed to be denying their true feelings, as endorsed by 61.8% of the sample—reinforces the notion that grief unfolds in a uniform manner for everyone. This view increases the risk of pathologizing responses that deviate from normative expectations about how people should feel and behave in the face of loss. Compared with the general population sample examined by Sawyer et al. (2022), in which slightly more than one quarter of participants considered this statement probably or definitely true, participants in the present study provided less accurate responses. Previous research has highlighted that bereavement is shaped by a wide range of factors (Buur et al., 2024; Mason et al., 2020), including attachment style, nature and significance of the bond with the deceased (Root & Exline, 2014), quality of the caregiving or dying experience (Garrido & Prigerson, 2014), nature and circumstances of the death (Kaplow et al., 2014), quality of support received (Tsai et al., 2016), and cultural norms (Irish et al., 2014; Silverman et al., 2021). Greater awareness of the factors that make each grief experience unique and idiosyncratic may help informal support providers respond to bereaved individuals with greater sensitivity and without judgment.

The belief that pain responses are typically consistent even when cultural differences are considered—endorsed by 65.7% of the present sample and by just over half of the general population sample in the study by Sawyer et al. (2022)—represents another way of overlooking the idiosyncratic nature of grief. Greater awareness of cultural differences could help teachers and health professionals better assess the types of support bereaved individuals may need. When there is uncertainty about religious or cultural preferences, adopting a stance of curiosity and asking respectful questions can be a meaningful way of expressing attention and care (Venuleo et al., 2022).

Other beliefs that similarly neglect the idiosyncratic nature of grief include the assumptions that *“elderly people are usually more anxious about death than young people”* and that *“bereaved children and adolescents do not grieve as deeply as adults,”* which were endorsed by 72.5% and 44.6% of the sample, respectively. In both cases, these percentages are higher than those reported by Sawyer et al. (2022), in which fewer than half and approximately one quarter of the general population sample endorsed these myths, respectively. Primary reference adults in children’s lives (e.g., family members and teachers) should be made aware that the death of a close person (such as a parent) represents a highly stressful psychological and social event for children. Children may express their grief in ways that differ from those of adults (Ferow, 2019); for example, they may communicate their distress through behaviour and play, a decline in school performance and sleep disturbances.

Other false beliefs concern the management of grief. The idea that *“it is generally more helpful for people to ‘move on’ with their lives rather than think about memories of the deceased”*—which was shared by only a minority of the general population sample in Sawyer et al. (2022)—as widely endorsed by respondents in the current study (71.6%). This myth can be highly detrimental, as it may promote unhelpful forms of support. Bereaved individuals may be encouraged to repress their thoughts and feelings (e.g., not crying, suffering in silence, appearing “strong”) and may judge themselves—or be judged by others—as “weak,” “crazy,” or “self-pitying” if they are unable to overcome their pain. Many bereaved people report feeling abandoned by family and friends who believe they have failed to move on in a timely manner (Doka, 2016). This belief is also critical because it may foster a fear among the bereaved that “moving on” comes at the expense of fading memories of the deceased (Doka, 2016). From the perspective of informal support providers, it may lead them to avoid mentioning the deceased or to distract the bereaved when they recall memories of their loved one. Greater awareness of the healing value of maintaining a living inner connection with the deceased would help informal support providers to acknowledge and share feelings related to the person who has died. This is particularly relevant for healthcare professionals working in hospital and nursing home settings who may have cared for the dying person over an extended period. For bereaved individuals, hearing anecdotes from caregivers about their loved one during the care period—such as how the deceased left a mark on staff in their final days—is a significant source of comfort (Menichetti-Delor et al., 2021; Venuleo et al., 2022). Family members want reassurance that their loved one mattered, that they were not merely a “number” to those who assisted them, and that healthcare professionals remember them in their uniqueness (Lichtenthal et al., 2020).

The idea that children should be protected from bereavement—endorsed by 60.1% of our sample—needs to be critically reconsidered. Children are often left to grieve alone by adults who are uncertain about the “right” thing to do or say (Duncan, 2020). However, concealing grief within family contexts or avoiding any reference to the loss at school communicates to children that it is inappropriate or unwelcome to talk about their experiences. Encouraging children to express and share emotions related to the loss is essential (Fearnley & Boland, 2017), and this responsibility extends beyond parents to other adults in their daily lives. Schools, where children spend a significant portion of their time, can play a crucial role in supporting bereaved students (Stylianou & Zembylas, 2018). Appropriate responses from teachers can positively influence both the short-term functioning and long-term adjustment of grieving children (Cohen & Mannarino, 2011). Nilsson and Ängarne-Lindberg (2016) suggested that children often speak about their grief with someone who is not directly affected by the loss. Immediately following the death of a family member, surviving relatives may struggle to support one another due to fears of overwhelming each other with grief. Friends may avoid mentioning the deceased or the bereaved entirely, sometimes encouraging them to be happy prematurely (Dickson, 2002). Teachers—and by extension, other child educators such as sports coaches and catechists—frequently represent the only adults positioned to provide guidance and support to grieving children. They can facilitate the child’s return to shared activities and create opportunities for expressing questions, feelings, and concerns through drawing, writing, or play. Peer support is also valuable as a coping mechanism to mitigate the social isolation caused by loss (Duncan, 2020; Ellis et al., 2013). However, peers often lack the knowledge and skills to effectively support grieving friends (Stylianou & Zembylas, 2018). Investments in education about death and dying are crucial, as these topics are often absent from school curricula (Duncan, 2020; Gunn, 2009). Facilitating group discussions on loss, including through books (Milton, 2004), and encouraging children to share personal experiences (including pet loss), validate their emotions, discuss received or desired support, and explore how they might help friends experiencing grief can normalize grief responses, reduce feelings of isolation, and allow children to observe and learn coping strategies from teachers and peers. This approach can also foster a supportive network among children facing similar experiences (Duncan, 2020; Venuleo et al., 2022).

The beliefs that most people develop a mental disorder after the death of a loved one and that most bereaved individuals need a psychologist were endorsed by 36.9% and 72.5% of our sample, respectively. In both cases, these percentages are higher than those reported in the study by Sawyer et al. (2022), where the myths were shared by 24% and 39% of the general population sample, respectively. Several scholars have highlighted the contemporary tendency to pathologize grief reactions (Breen & O’Connor, 2007; Granek, 2008), which may contribute to the perception of mental health professionals as the primary source of support for bereaved individuals. A recent qualitative study also indicated that informal support providers tend to underestimate the role that each community member can play in supporting a bereaved person (Marinaci et al., 2024). However, research consistently shows that most people are able to navigate grief successfully with the support of a compassionate social network, including family, friends, teachers, healthcare providers, and other community members (Aoun et al., 2018). Health training programs should aim to improve knowledge of common and complicated grief reactions, helping support providers recognize when professional intervention is necessary (Sawyer et al., 2022). Equally important is fostering awareness of the critical role that each member of a bereaved individual’s social network can play in promoting adaptive responses to loss.

Significant differences across respondents’ roles were found for only two of the nine myths. Health and social care professionals provided more accurate responses than teachers and the general population regarding the myths that “*elderly people are usually more anxious about death than young people*” and that “*bereaved children and adolescents do not grieve as deeply as adults.*” This difference may be explained by direct experience in care and treatment settings or by knowledge acquired during professional training in social and health fields. These findings underscore the need to raise awareness among family members, friends, and teachers—who often serve as primary support for grieving children—about how children also experience and express the painful emotions associated with bereavement. The absence of other significant role-related differences highlights the substantial educational gap on the topic of loss and bereavement that, in the Italian context of this study, also affects teachers and social-health professionals. The COVID-19 pandemic has further emphasized that preventing complicated bereavement reactions is a collective responsibility, requiring the promotion of grief literacy and supportive skills among social and health workers, teachers, and all members of the community who interact with bereaved individuals as they navigate the difficult process of accepting and making sense of loss (Venuleo et al., 2022).

Regarding sex-related differences, women were more likely than men to consider grief a linear process, but at the same time, they provided more accurate responses than men when evaluating the statement that “*bereaved children and adolescents do not grieve as deeply as adults*.” Previous studies in American samples (Sexton, 2013; Tatar, 2017) found that women endorsed myths more strongly than men. One possible explanation is that women often occupy caregiving roles, which may make them more aware of child bereavement and simultaneously more likely to seek information about the grieving process, exposing them to misconceptions and grey literature circulating in social networks.

No age-related differences were found in the present study, in contrast to Tatar (2017), who reported that older participants provided more inaccurate responses than younger ones. The author suggested that age differences in myth endorsement likely reflect the degree to which scientific notions are publicly debated. It is possible that the information available in Italy is less up to date with recent empirical evidence.

Finally, it is noteworthy that having experienced a significant loss appears to protect against misconceptions regarding grief. Participants who had suffered a significant loss were less likely to endorse the myths that most bereaved people develop a mental disorder, that people who do not become depressed are likely denying their true feelings, and that it is more useful to “move on” with one’s life rather than thinking about the memories of the deceased. Conversely, participants without such experience may have relied more heavily on advice and commentary received from their social network when forming beliefs about grief.

The high levels of endorsement of grief-related myths observed across roles and demographic groups in our sample should be interpreted not only in light of individual knowledge gaps but also within the broader socio-cultural context previously outlined. As noted, historical shifts in Italian mourning practices and the gradual reduction in collectively shared guidance on grief may have created conditions in which simplified psychological models continue to influence public and professional understandings. This perspective reinforces the need to strengthen community-based support and grief literacy, highlighting the role that social networks and everyday contacts can play in fostering adaptive bereavement responses.

### Limitations

This study has several methodological limitations. The relatively small convenience sample limits the generalizability of the results. In addition, the recruitment method may have introduced self-selection bias, as individuals who chose to participate could be more interested in grief-related topics or have previous personal or professional exposure to bereavement, which may not represent the broader population of informal support providers. In Italy, the context of this study, there are very few training initiatives on loss and bereavement, which may partly explain the widespread adherence of respondents to the proposed myths. Other factors potentially associated with participants’ competencies, such as prior participation in grief-related training, were not assessed. Future research should explore whether these findings are applicable in other cultural contexts and examine the effects of targeted training programs on informal support providers’ understanding of grief and the needs of bereaved individuals.

## 5. Conclusions

This study highlights widespread misconceptions about grief and bereavement among informal support providers in Italy, including health and social care professionals, teachers, and the general population. Misbeliefs concerning the linearity of grief, the necessity of professional help, and age- or culture-related grief responses were particularly prevalent. Enhancing awareness of the diversity, developmental, and cultural dimensions of grief and recognising the legitimacy of varied grief reactions can help informal support providers deliver more effective, empathetic, and informed support to bereaved individuals. Strengthening these competencies is especially relevant in the aftermath of the COVID-19 pandemic, which underscored the collective responsibility of communities to support those experiencing loss.

## Figures and Tables

**Table 1:** Participants’ demographic characteristics.

Variable		F (%)	χ^2^ (df)
Sex ^1^	Man	89 (38.2)	12.160 (1) ***
	Woman	142 (60.9)	
Age range	18–29	56 (24.0)	51.240 (3) ***
	30–44	62 (26.6)	
	45–59	96 (41.2)	
	Over 60	19 (8.2)	
Educational status	Middle school	8 (3.4)	131.584 (3) ***
	High school	108 (46.4)	
	Bachelor’s degree	95 (40.8)	
	Post bachelor’s degree	22 (9.4)	
Loss experience	Yes	189 (81.1)	90.236 (1) ***
	No	44 (18.9)	

*** *p* < 0.001; df = Degrees of Freedom; ^1^ Missing value = 2 (0.9).

**Table 2:** Frequency and percentage of responses to grief myths reported by the three groups (health and social care professionals, teachers and educators, and the general population).

		Frequency (%)		
Myths	Response	Health and Social Care Professionals	Teachers	General Population
Grieving can be expected to progress through a predictable series of stages. beginning with denial and ending with acceptance	**Definitely false**	1 (1.6)	1 (1.2)	1 (1.1)
	**Probably false**	1 (1.6)	2 (2.4)	6 (7.0)
	Probably true	26 (42.0)	46 (54.7)	47 (54.0)
	Definitely true	34 (54.8)	35 (41.7)	33 (37.9)
2. People who do not become depressed after the death of a loved one are likely to denying their true feelings	**Definitely false**	8 (12.9)	18 (21.4)	7 (8.0)
	**Probably false**	15 (24.2)	17 (20.2)	24 (27.6)
	Probably true	23 (37.1)	39 (46.4)	44 (50.6)
	Definitely true	16 (25.8)	10 (12.0)	12 (13.8)
3. Most people develop a mental disorder after the death of a loved one	**False**	21 (33.9)	35 (41.7)	23 (26.4)
	**Probably false**	16 (25.8)	23 (27.4)	29 (33.3)
	Probably true	19 (30.6)	21 (25.0)	29 (33.3)
	Definitely true	6 (9.7)	5 (5.9)	6 (7.0)
4. Pain responses are typically consistent even when cultural differences are considered	**Definitely false**	11 (17.7)	10 (11.9)	10 (11.5)
	**Probably false**	8 (12.9)	17 (20.2)	24 (27.6)
	Probably true	23 (37.1)	42 (50.0)	34 (39.1)
	Definitely true	20 (32.3)	15 (17.9)	19 (21.8)
5. Elderly people are usually more anxious about death than young people	**Definitely false**	7 (11.3)	5 (5.9)	3 (3.5)
	**Probably false**	18 (29.0)	16 (19.1)	15 (17.2)
	Probably true	23 (37.1)	48 (57.1)	49 (56.3)
	Definitely true	14 (22.6)	15 (17.9)	20 (23.0)
6. Bereavement children and adolescents do not grieve as deeply as adults	**Definitely false**	27 (43.5)	30 (35.7)	19 (21.8)
	**Probably false**	16 (25.8)	20 (23.8)	17 (19.5)
	Probably true	13 (21.0)	22 (26.2)	32 (36.8)
	Definitely true	6 (9.7)	12 (14.3)	19 (21.8)
7. Generally, it is more useful to ‘move on’ with one’s life rather than thinking about the memories of the deceased	**Definitely false**	10 (16.1)	11 (13.1)	4 (4.6)
	**Probably false**	13 (21.0)	9 (10.7)	19 (21.8)
	Probably true	26 (41.9)	50 (59.5)	43 (49.4)
	Definitely true	13 (21.0)	14 (16.7)	21 (24.1)
8. Experts generally recommend that children be protected from the pain and suffering created by death	**False**	10 (16.1)	9 (10.7)	9 (10.3)
	**Probably false**	21 (33.9)	20 (23.8)	24 (27.6)
	Probably true	14 (22.6)	43 (51.2)	33 (38.0)
	Definitely true	17 (27.4)	12 (14.3)	21 (24.1)
9. Most people need professional help to deal with pain	**False**	5 (5.9)	1 (1.6)	8 (9.2)
	**Probably false**	22 (26.2)	11 (17.7)	17 (19.5)
	Probably true	42 (50.0)	23 (37.1)	45 (51.7)
	Definitely true	15 (17.9)	27 (43.6)	17 (19.5)

Note: Responses that, based on recent literature, are considered accurate (“definitely false” or “probably false”) are highlighted in bold. Health and Social Care Professionals: n = 62 (women: 75.8%), Mean Age = 40.1 (SD = 11.1); Teachers: n = 84 (women: 69%), Mean Age = 44.5 (SD = 13.2); General Population: n = 87 (women: 42.5%), Mean Age = 41.6 (SD = 13.1).

**Table 3:** Frequency and chi-square results for grief myths reported by the three groups (health and social care professionals, teachers and educators, and the general population).

Myths	Response	Frequency (%)				χ^2^ (df = 1)
		Overall Sample	Health and Social Care Professional	Teachers	General Population	
Grieving can be expected to progress through a predictable series of stages. beginning with denial and ending with acceptance	Accurate responses	12 (5.2)	2 (3.2)	3 (3.6)	7 (8.1)	2.392
	Inaccurate responses	221 (94.8)	60 (96.8)	81 (96.4)	80 (91.9)	
2. People who do not become depressed after the death of a loved one are likely to denying their true feelings	Accurate responses	89 (38.2)	23 (37.1)	35 (41.7)	31 (35.6)	0.703
	Inaccurate responses	144 (61.8)	39 (62.9)	49 (58.3)	56 (64.4)	
3. Most people develop a mental disorder after the death of a loved one	Accurate responses	147 (63.1)	37 (59.7)	58 (69.0)	52 (59.8)	2.002
	Inaccurate responses	86 (36.9)	25 (40.3)	26 (31.0)	35 (40.2)	
4. Pain responses are typically consistent even when cultural differences are considered	Accurate responses	80 (34.3)	19 (30.6)	27 (32.1)	34 (39.1)	1.422
	Inaccurate responses	153 (65.7)	43 (69.4)	57 (67.9)	53 (60.9)	
5. Elderly people are usually more anxious about death than young people	Accurate responses	64 (27.5)	25 (40.3)	21 (25.0)	18 (20.7)	7.405 *
	Inaccurate responses	169 (72.5)	37 (59.7)	63 (75.0)	69 (79.3)	
6. Bereavement children and adolescents do not grieve as deeply as adults	Accurate responses	129 (55.4)	43 (69.4)	50 (59.5)	36 (41.4)	12.384 **
	Inaccurate responses	104 (44.6)	19 (30.6)	34 (40.5)	51 (58.6)	
7. Generally, it is more useful to ‘move on’ with one’s life rather than thinking about the memories of the deceased	Accurate responses	66 (28.3)	23 (37.1)	20 (23.8)	23 (26.4)	3.346
	Inaccurate responses	167 (71.7)	39 (62.9)	64 (76.2)	64 (73.6)	
8. Experts generally recommend that children be protected from the pain and suffering created by death	Accurate Responses	93 (39.9)	31 (50.0)	29 (34.5)	33 (37.9)	3.790
	Inaccurate responses	140 (60.1)	31 (50.0)	55 (65.5)	54 (62.1)	
9. Most people need professional help to deal with pain	Accurate responses	64 (27.5)	12 (19.4)	27 (32.1)	25 (28.7)	3.040
	Inaccurate responses	169 (72.5)	50 (80.6)	57 (67.9)	62 (71.3)	

Note: Accurate responses: Sum of definitely and probably false responses—Inaccurate responses: Sum of definitely true and probably true responses; * *p* < 0.05; ** *p* < 0.01; df = Degrees of Freedom; *Overall sample*: n = 233 (woman: 60.9%), Mean Age = 42.2 (SD = 12.7); Health and Social Care Professional: n = 62 (women: 75.8%); Mean Age = 40.1 (SD = 11.1); Teachers: n = 84 (woman: 69%), Mean Age = 44.5 (SD = 13.2); General Population: n = 87 (woman: 42.5%), Mean Age = 41.6 (SD = 13.1). In the total sample (n = 233), 26.6% of participants were health and social care professionals, 36.5% were teachers, and 37.3% were from the general population.

**Table 4:** Frequencies, percentages of accurate and inaccurate responses, and chi-square results for grief myths according to participants’ socio-demographic characteristics.

			Myth 1	Myth 2	Myth 3	Myth 4	Myth 5	Myth 6	Myth 7	Myth 8	Myth 9
Sex	Man	Accurate responses	9 (10.1)	31 (34.8)	60 (67.4)	31 (34.8)	25 (28.1)	40 (44.9)	26 (29.2)	29 (32.6)	27 (30.3)
		Inaccurate responses	80 (89.9)	58 (65.2)	29 (32.6)	58 (65.2)	64 (71.9)	49 (55.1)	63 (70.8)	60 (67.4)	62 (69.7)
	Woman	Accurate responses	3 (2.1)	56 (39.4)	85 (59.9)	48 (33.8)	39 (27.5)	87 (61.3)	39 (27.5)	62 (43.7)	36 (25.4)
		Inaccurate responses	139 (97.9)	86 (60.6)	57 (40.1)	94 (66.2)	103 (72.5)	55 (38.7)	103 (72.5)	80 (56.3)	106 (74.6)
	χ^2^ (df = 2)		7.277 *	3.755	2.522	0.245	0.776	7.526 *	0.549	5.836	1.196
Age range	18–29	Accurate responses	3 (5.4)	20 (35.7)	28 (50.0)	16 (28.6)	16 (28.6)	30 (46.4)	14 (25.0)	18 (32.1)	11 (19.6)
		Inaccurate responses	53 (94.6)	36 (64.3)	28 (50.0)	40 (71.4)	40 (71.4)	26 (53.6)	42 (75.0)	38 (67.9)	45 (80.4)
	30–44	Accurate responses	1 (1.6)	24 (38.7)	43 (69.4)	27 (43.6)	20 (32.3)	41 (66.1)	22 (35.5)	25 (40.3)	18 (29.0)
		Inaccurate responses	61 (98.4)	38 (61.3)	19 (30.6)	35 (56.4)	42 (67.7)	21 (33.9)	40 (64.5)	37 (59.7)	44 (71.0)
	45–59	Accurate responses	7 (7.3)	38 (39.6)	62 (64.6)	30 (31.3)	26 (27.1)	51 (53.1)	27 (28.1)	42 (43.7)	28 (29.2)
		Inaccurate responses	89 (92.7)	58 (60.4)	34 (35.4)	66 (68.7)	70 (72.9)	45 (46.9)	69 (71.9)	54 (56.2)	68 (70.8)
	Over 60	Accurate responses	1 (5.3)	7 (36.8)	14 (73.7)	7 (36.8)	2 (10.5)	7 (36.8)	3 (15.8)	8 (42.1)	7 (36.8)
		Inaccurate responses	18 (94.7)	12 (63.2)	5 (26.3)	12 (63.2)	17 (89.5)	12 (63.2)	16 (84.2)	11 (57.9)	12 (63.2)
	χ^2^ (df = 3)		2.495	0.246	6.173	3.618	3.493	5.813	3.342	2.042	2.774
Educational status	Middle school	Accurate responses	0 (0.0)	3 (37.5)	4 (50.0)	3 (37.5)	1 (12.5)	3 (37.5)	2 (25.0)	4 (50.0)	7 (12.5)
		Inaccurate responses	8 (100.0)	5 (62.5)	4 (50.0)	5 (62.5)	7 (87.5)	5 (62.5)	6 (75.0)	5 (50.0)	7 (87.5)
	High school	Accurate responses	7 (6.5)	39 (36.1)	62 (57.4)	35 (32.4)	25 (23.1)	51 (47.2)	28 (25.9)	37 (34.3)	28 (25.9)
		Inaccurate responses	101 (93.5)	69 (63.9)	46 (42.6)	73 (67.6)	83 (76.9)	57 (52.8)	80 (74.1)	71 (65.7)	80 (74.1)
	Bachelor’s Degree	Accurate responses	5 (5.3)	40 (42.1)	6 (70.5)	37 (39.0)	33 (34.7)	61 (64.2)	5 (22.7)	43 (45.3)	27 (28.4)
		Inaccurate responses	90 (94.7)	55 (57.9)	28 (29.5)	58 (61.0)	62 (65.3)	34 (35.8)	17 (77.3)	52 (54.7)	68 (71.6)
	Post Bachelor’s Degree	Accurate responses	0 (0.0)	7 (31.8)	14 (63.6)	5 (22.7)	5 (22.7)	3 (37.5)	31 (32.6)	9 (40.9)	8 (36.4)
		Inaccurate responses	22 (100.0)	15 (68.2)	8 (36.4)	17 (77.3)	17 (77.3)	5 (62.5)	64 (67.4)	13 (59.1)	14 (63.6)
	χ^2^ (df = 3)		2.023	1.195	4.345	2.425	4.679	7.548	1.557	2.992	1.946
Loss experience	Yes	Accurate responses	10 (5.3)	79 (41.8)	125 (66.1)	67 (35.5)	54 (28.6)	109 (57.7)	59 (31.2)	81 (42.9)	51 (27.0)
		Inaccurate responses	179 (94.7)	110 (58.2)	64 (33.9)	122 (64.6)	135 (71.4)	57 (42.3)	130 (68.8)	108 (57.1)	138 (73.0)
	No	Accurate responses	2 (4.6)	10 (22.7)	22 (50.0)	13 (29.5)	10 (22.7)	20 (45.5)	7 (15.9)	12 (27.3)	13 (29.5)
		Inaccurate responses	42 (95.4)	34 (77.3)	22 (50.0)	31 (70.5)	34 (77.3)	24 (54.5)	37 (84.1)	32 (72.7)	31 (70.5)
	χ^2^ (df = 1)		0.041	5.499 *	3.991 *	0.552	0.612	2.156	4.119 *	3.614	0.118

Note: Accurate responses: Sum of definitely and probably false responses—Inaccurate responses: Sum of definitely true and probably true response; df = Degrees of Freedom; Myth 1: Grieving can be expected to progress through a predictable series of stages, beginning with denial and ending with acceptance; Myth 2: People who do not become depressed after the death of a loved one are likely to denying their true feelings; Myth 3: Most people develop a mental disorder after the death of a loved one; Myth 4: Pain responses are typically consistent even when cultural differences are considered; Myth 5: Elderly people are usually more anxious about death than young people; Myth 6: Bereavement children and adolescents do not grieve as deeply as adults; Myth 7: Generally, it is more useful to ‘move on’ with one’s life rather than thinking about the memories of the deceased; Myth 8: Experts generally recommend that children be protected from the pain and suffering created by death. Myth 9: Most people need professional help to deal with pain. * *p* < 0.05.

## Data Availability

The original data presented in the study are openly available in Zenodo at https://doi.org/10.5281/zenodo.18388154. The dataset is fully anonymized and released under a Creative Commons Attribution 4.0 International (CC BY 4.0) license.
